# Dynamic Computed Tomography Angiography for capturing vessel wall motion: A phantom study for optimal image reconstruction

**DOI:** 10.1371/journal.pone.0293353

**Published:** 2023-12-22

**Authors:** Lotte B. Stam, Sabine M. L. Linden, Luuk J. Oostveen, Hendrik H. G. Hansen, René Aquarius, Cornelis H. Slump, Chris L. de Korte, Ronald H. M. A. Bartels, Mathias Prokop, Hieronymus D. Boogaarts, Frederick J. A. Meijer

**Affiliations:** 1 Department of Neurosurgery, Radboudumc, Nijmegen, The Netherlands; 2 Technical Medical Center, University of Twente, Enschede, The Netherlands; 3 Department of Medical Imaging, Radboudumc, Nijmegen, The Netherlands; Medical University of Vienna: Medizinische Universitat Wien, AUSTRIA

## Abstract

**Background:**

Reliably capturing sub-millimeter vessel wall motion over time, using dynamic Computed Tomography Angiography (4D CTA), might provide insight in biomechanical properties of these vessels. This may improve diagnosis, prognosis, and treatment decision making in vascular pathologies.

**Purpose:**

The aim of this study is to determine the most suitable image reconstruction method for 4D CTA to accurately assess harmonic diameter changes of vessels.

**Methods:**

An elastic tube (inner diameter 6 mm, wall thickness 2 mm) was exposed to sinusoidal pressure waves with a frequency of 70 beats-per-minute. Five flow amplitudes were set, resulting in increasing sinusoidal diameter changes of the elastic tube, measured during three simulated pulsation cycles, using ECG-gated 4D CTA on a 320-detector row CT system. Tomographic images were reconstructed using one of the following three reconstruction methods: hybrid iterative (Hybrid-IR), model-based iterative (MBIR) and deep-learning based (DLR) reconstruction. The three reconstruction methods where based on 180 degrees (half reconstruction mode) and 360 degrees (full reconstruction mode) raw data. The diameter change, captured by 4D CTA, was computed based on image registration. As a reference metric for diameter change measurement, a 9 MHz linear ultrasound transducer was used. The sum of relative absolute differences (SRAD) between the ultrasound and 4D CTA measurements was calculated for each reconstruction method. The standard deviation was computed across the three pulsation cycles.

**Results:**

MBIR and DLR resulted in a decreased SRAD and standard deviation compared to Hybrid-IR. Full reconstruction mode resulted in a decreased SRAD and standard deviations, compared to half reconstruction mode.

**Conclusions:**

4D CTA can capture a diameter change pattern comparable to the pattern captured by US. DLR and MBIR algorithms show more accurate results than Hybrid-IR. Reconstruction with DLR is >3 times faster, compared to reconstruction with MBIR. Full reconstruction mode is more accurate than half reconstruction mode.

## 1. Introduction

Dynamic Computed Tomography Angiography (4D CTA) allows noninvasive evaluation of vessel dynamics by the acquisition of multiple CT scans over time [1]. Whereas a single phase CTA enables the evaluation of the anatomy and dimensions of vessels, 4D CTA facilitates evaluation of extension and shape changes of vessels over each cardiac cycle. Vessel wall motion is a result of the hemodynamic stress on the vessel wall caused by the propagating cardiac pulse pressure wave and the biomechanical properties of the vessel wall. Vessel wall motion may serve as a surrogate marker for decreased vessel wall stability, which could provide valuable insights for vascular pathologies, such as cerebral aneurysms, or cardiovascular diseases [2,3]. Therefore, insight in vessel wall motion might provide information on the biomechanical properties of these vessels and may subsequently improve diagnosis, prognosis and treatment decision for different vascular pathologies.

More specific, it has been reported that wall motion of cerebral aneurysms uncovers important information about the stability and thus for rupture risk estimation [4,5]. Refinement of the rupture risk estimation may improve selection of patients who are likely to benefit from preventative treatment, despite the complication risks associated with treatment. A relative vessel diameter change of 10% may sound substantial, in case of an aneurysm with a diameter of 3 mm, this only results in an absolute change of 0.3 mm [6]. Therefore, it is challenging for current non-invasive imaging systems to capture sub-millimetric vessel diameter changes and intracranial aneurysm wall motion, especially when the patient has a high heart rate [7].

In literature, only one study has reported on the accuracy of 4D CTA for capturing vessel wall movement. A previous pilot study evaluated cerebral aneurysm volume changes, showing the ability of 4D CTA to capture volume changes of approximately 3 mm^3^, where a sinusoidal pressure wave of 90 bpm was applied to a spherical phantom. [8] This study related the measured expansion to the input settings, instead of using a reference imaging technique. A validation study for 4D CTA for capturing vessel movement using a phantom with a reference measurement is lacking in literature.

A reference measurement with a superior spatial and temporal resolution compared to 4D CTA is needed to estimate the performance of 4D CTA. Echo-tracking based on ultrasound (US) captures vessel wall motion with a spatial resolution up to 1 μm and has a sufficient temporal resolution to capture cardiac-cycle related motion. [9] Echo-tracking is based on raw frequency (RF) data and uses a normalized cross-correlation-based algorithm for vessel wall tracking. [10] US can be used as reliable reference in a phantom experiment.

The quality of 4D CTA scans depends on the acquisition parameters and the image reconstruction methods. Given the ultimate objective to detect subtle vessel wall motion, a critical determination revolves around identifying the most effective image reconstruction method for subsequent analysis. The purpose of this phantom study is to determine the most optimal image reconstruction method for 4D CTA in order to accurately capture harmonic diameter changes of vessels.

## 2. Materials and methods

### 2.1 Experimental set-up

**Fig 1:**
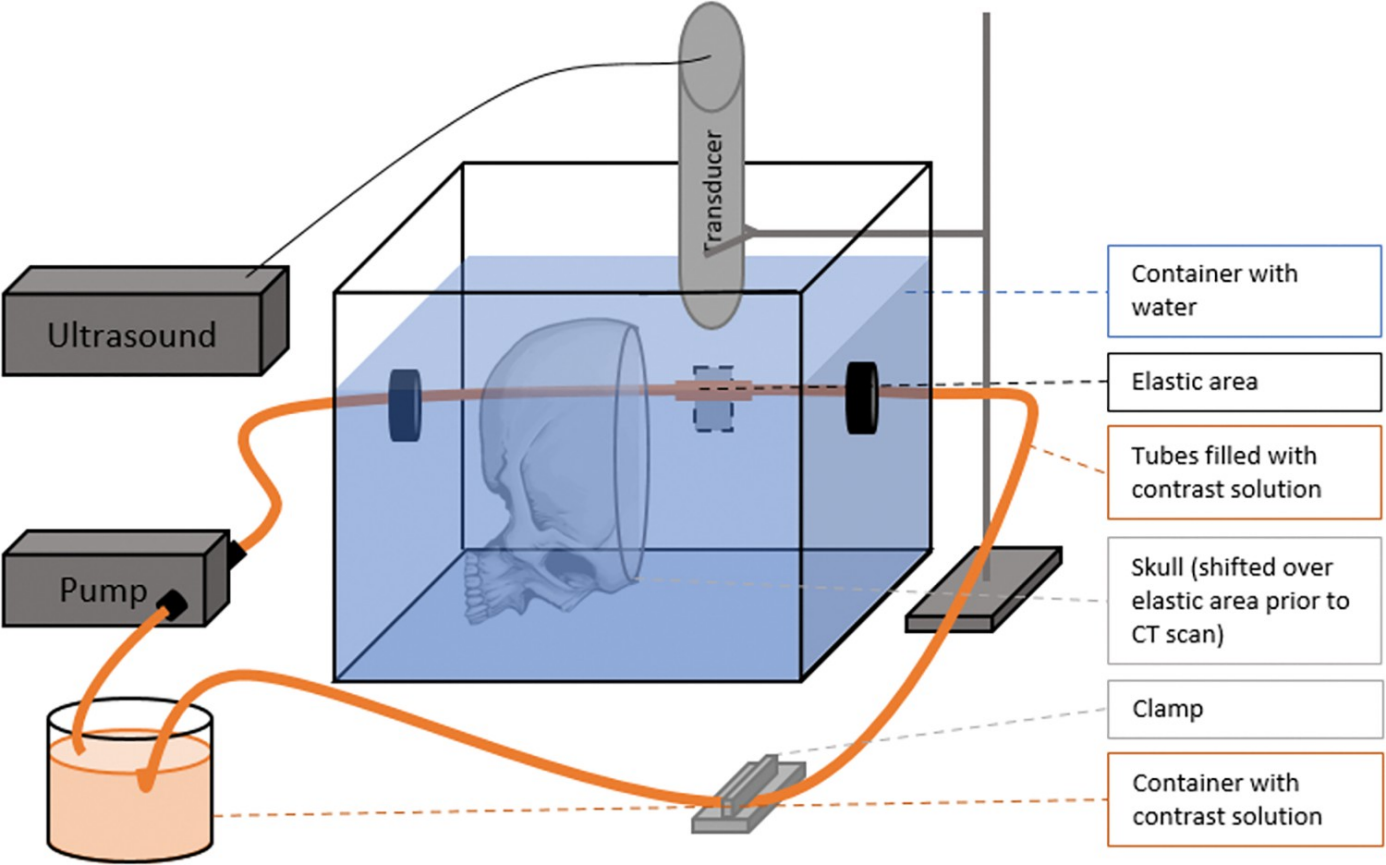
Schematic representation of the experimental set-up for the ultrasound measurement (the skull is moved away from the elastic tube). The polymethylmethacrylate container, with a size of 21x21x15 cm (length x width x height), was filled with demineralized water at room temperature.

A schematic representation of the experimental set-up is shown in ***Fig 1.*** An elastic tube *(Fluid Transfer Versilic Flexible Tubing Silicone hardness 62 (Shore A)*, *Saint Gobain*, *Courbevoie*, *France)* with a length of approximately 4 cm, a luminal diameter of 6 mm and a wall thickness of 2 mm, was connected to rigid, polyethylene tubes (*Norgren LCC*, *Littleton*, *USA)* with a luminal diameter of 6 mm and a wall thickness of 1 mm. The circuit was filled with a diluted iodinated contrast solution of 12.5 mg I/ml *(Iomeron 300*, *Bracco*, *Konstanz*, *Germany)*. This concentration resulted in density levels visually comparable to the density levels of contrast enhanced blood in in vivo CT angiography, under the specific scan parameters as listed in section 2.3. The elastic tube was placed in a container filled with water and a human skull was placed around the elastic tube during 4D CTA acquisition to mimic x-ray attenuation caused by bone. The skull was moved away from the elastic tube to allow for unhindered US acquisition.

A sinusoidal flow wave with a frequency of 1.17 Hz, corresponding to 70 beats-per-minute (bpm), was created with a flow-regulating pump (*model H3F*, *Liquiflo*, *Garwood*, *USA*). The baseline flow of 0.3 L/min was combined with five additional sinusoidal flow amplitudes:

- Setting 1: 0.1 L/min
- Setting 2: 0.3 L/min
- Setting 3: 0.5 L/min
- Setting 4: 0.7 L/min
- Setting 5: 0.9 L/min

This alternating flow created an alternating pressure due to the placement of a clamp at the end of the circuit. The alternating pressure resulted in a periodic expansion of the elastic segment with a magnitude depending on the flow amplitude of the pump.

### 2.2 Ultrasound

All US acquisitions were done before 4D CTA acquisition.

#### 2.2.1 Acquisition

The US transducer was placed in a mechanical holder above the elastic tube and faced downwards. The reflection from vessel wall to blood was adjusted to align the vessel wall with the central plan of the ultrasound probe, based on visual inspection of the reflected wave. Raw radiofrequency (RF) US data of the tube in a transverse view were recorded for 3 seconds using a 9 MHz (L5–13) linear array transducer, which enabled detection of three sinusoidal wave forms. The transducer was connected to an Accuvix V10 ultrasound system *(Samsung Medison*, *Seoul*, *South Korea)* equipped with an interface providing raw RF ultrasound data at a sampling frequency of 61.6 Hz. The line density was 200 μm.

#### 2.2.2 Postprocessing

The RF data were imported in Matlab (Version R2018a, The Mathworks Inc, Natick, Massachusetts, USA) and analyzed using an in-house developed software tool, as described in ***S1 Appendix.*** This tool analyses the raw radiofrequency ultrasound data. A user selects the reflections in one time frame and the motion of these reflections is automatically determined by a 2D normalized cross-correlation-based algorithm [10].

### 2.3 4D CTA

#### 2.3.1 Acquisition

The 4D CTA scans were performed, using a 320-detector row CT system *(Aquilion ONE Prism Edition Canon Medical Systems*, *Otawara*, *Japan)*. The elastic tube surrounded by the skull was positioned in the centre of the gantry. Volume scans were made with a tube voltage of 100 kV, a tube current of 340 mA and a gantry rotation time of 0.28 s. Continuous sequential acquisitions with a collimation of 320 x 0.5 mm were performed for 3 pulsation cycles, while the ECG signal was recorded. The slice thickness was 0.5 mm, the slice increment 0.25 mm, the matrix size 512 x 512 and the data collection diameter 240 mm. An ECG signal with a frequency equal to the flow pump frequency was simulated and three consecutive pulsation cycles were scanned.

#### 2.3.2. Reconstruction

Per pulsation cycle, twenty datasets were reconstructed with retrospective ECG gating [11], ensuring that each dataset represented a time phase of 5% of one pulsation cycle. Half and full image reconstruction mode were combined with three image reconstruction algorithms:

- Hybrid Iterative Reconstruction (Hybrid-IR) *[12]*
- Model-based Iterative Reconstruction (MBIR) [13]
- Deep-learning based reconstruction (DLR) [14]

The corresponding reconstruction Field-Of-View (FOV) for each algorithm were selected as small as possible. Resulting in a FOV of 25 mm for Hybrid-IR, 51 mm for MBIR and 100 mm for DLR.

#### 2.3.3 Postprocessing

The computation of the diameter change for the CT acquired data was based on image registration, which is a method for aligning multiple images or volumes. Deformable registration, which is a type of image registration, deformed the voxels in order to align the volumes reconstructed at various time phases. Quantifying variations in voxel size across the time phases can offer valuable insights into alterations in vascular lumen size. The diameter change pattern was computed with the steps below:

1. The reconstructed CT datasets were imported in Matlab (Version R2018a, The Mathworks, Inc, Natick, Massachusetts, USA). In each dataset, a subvolume of the elastic tube with the length of 1 cm in longitudinal tube direction was manually selected, based on pixel size.
2. The voxel deformation was determined between the subvolumes of every time phase compared to the first volume of the corresponding pulsation cycle (0%), using MevisLab (Version 3.2a, MeVis Medical Solutions AG Bremen, Germany). Normalized Gradient Field (NGF) [15] was chosen as a distance measure. An edge parameter of 150 HU and a regularize value of 0.1 were chosen as parameter settings.
3. The alterations in vascular lumen size was determined by quantifying the voxel deformation. The mean determinant of the Jacobian [16] expressing the voxel deformation within the elastic tube (threshold = 130 HU) was computed. The influence of the value of this threshold was minimal, which can be seen in ***S2 Appendix*.** The volume change was converted into a diameter change by assuming the cylindric shape of the tube.

### 2.4 Alignment of 4D CTA and ultrasound diameter change

The cross correlation between the CT and the US signal was calculated, in order to align the phases of the US and CT diameter signal. First, the US signal was resampled to the corresponding sample frequency of the CT signal. An averaged CT pulsation cycle was calculated by averaging the diameter change over the three pulsation cycles. The cross correlation was calculated by shifting the averaged CT signal over the US signal. The shift corresponding with the highest value of the cross correlation coefficient was used to align the US signal with the CT signal.

### 2.5 Outcome measures

#### 2.5.1 Ultrasound: Maximal diameter changes

For each flow amplitude setting, the maximal diameter change was computed, measured with ultrasound. Thereby, the standard deviation on this maximal diameter change was calculated, based on the three highest peaks captured by ultrasound.

#### 2.5.2 Ultrasound: Intra- and inter reader agreement

To study the effect of the manual selection of the reflections on the measured diameter change, two measurements were performed by the same reader with a four months interval, and additionally one by a second reader. The interclass correlation coefficients (ICC) were calculated as the degree of absolute agreement among measurements [17]. Given that US serves as the reference metric, achieving an excellent ICC (>0.9) [18] for both intra- and inter reader agreement is a requirement for the utilization of this approach. The ICC was estimated the for two purposes:

- The intra-reader agreement: determined by comparing the maximal diameter changes between two measurements performed by the same reader.
- The inter-reader agreement: determined by comparing the maximal diameter selected by both readers (L. S. and H. H.).

#### 2.5.3 CT reconstruction and postprocessing time

For each image reconstruction method, the reconstruction time was recorded. Additionally, the postprocessing time was recorded.

#### 2.5.4 Diameter change pattern

The highest amplitude (A = 0.9 L/min) was chosen to visualize the diameter change pattern. The averaged diameter change pattern of three pulsation cycles was visualized. The minimum and maximum of every time point was plotted as error bar, serving as a metric for variability across the pulsation cycles. The diameter change pattern measured by US was visualized for comparison.

#### 2.5.5 Difference between 4D CTA and US measurements

To quantitatively assess the difference between the diameter change patterns obtained from 4D CTA and US, a difference metric was computed. The sum of relative absolute differences (SRAD) between the diameter change pattern derived from US and 4D CTA was determined according to the following formula:

$$SRAD=\sum_{n=1}^{20}\frac{\left|D_{US,n}-D_{CT,n}\right|}{D_{US,n}}$$

**Fig 2:**
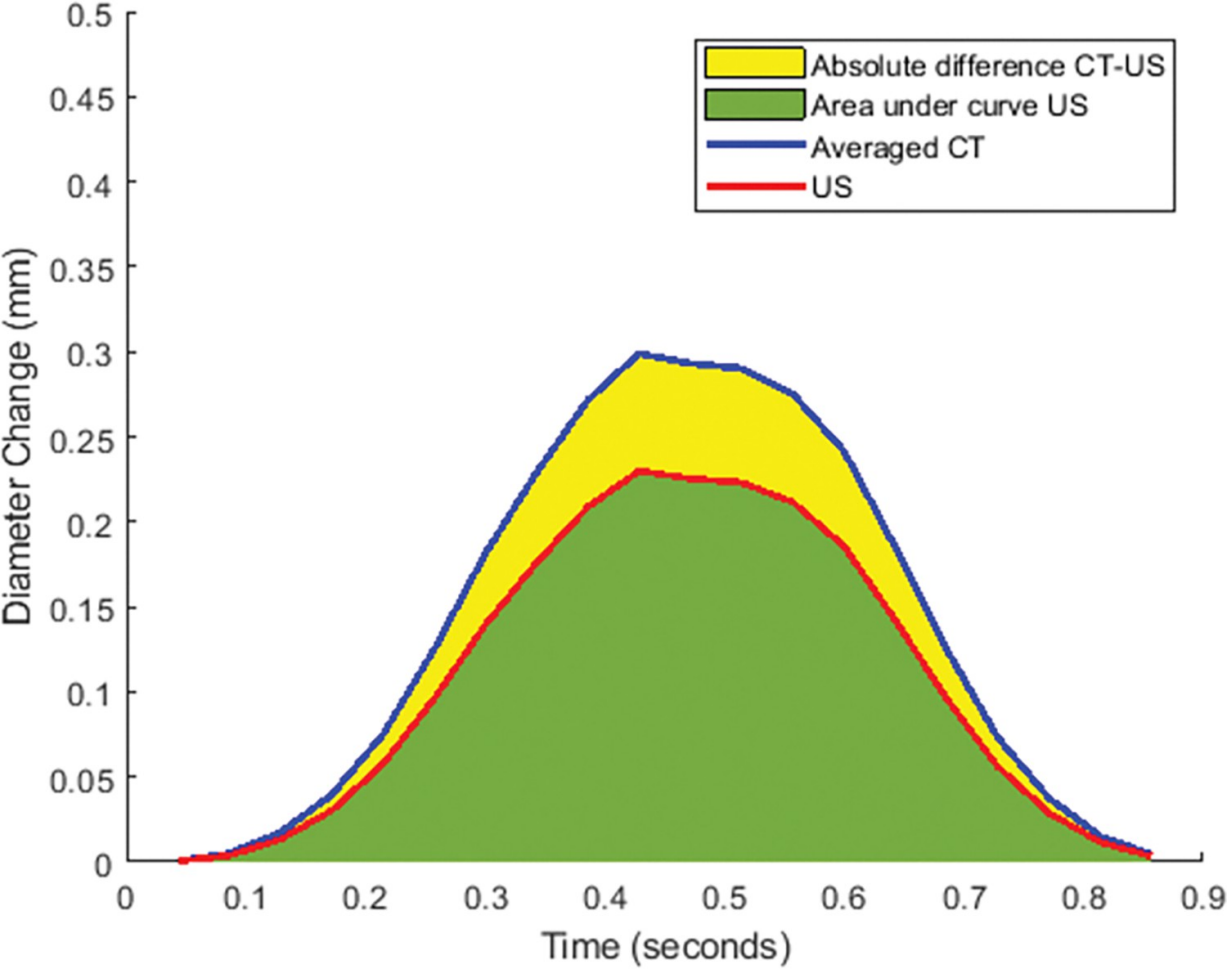
Visualization of SRAD.

D is the diameter change at time phase n, for CT and US. This metric represents the absolute difference between the area under the curve of the 4D CTA measurement and the area under the curve for the US measurement, for each pulsation cycle (**Fig 2**). When the diameter change measured by US (green) decreases, while the absolute difference between the between US and CT (yellow) remains constant, the SRAD increases.

The SRAD was computed for every pulsation cycle and the standard deviation was calculated across the pulsation cycles, serving as a metric for quantifying the variability among pulsation cycles.

## 3. Results

### 3.1 Ultrasound: Maximal diameter changes

The estimated diameter changes for the various applied flow amplitudes are listed in ***Table 1*** and varied between 0.008 and 0.265 mm (standard deviation varied between 0.001 and 0.004 mm). The diameter curves measured by ultrasound for each pump amplitude are shown in ***S3 Appendix***. The data used for this calculation can be found in ***S4 Appendix***.

**Table 1:** Mean diameter change and standard deviation per pulsation cycle measured with ultrasound for the different measurements. L = Liter, min = minutes, mm = millimeters.

Setting	Pump amplitude (L/min)	Mean diameter change (mm)	Standard deviation (mm)
1	0.1	0.008	0.001
2	0.3	0.035	0.004
3	0.5	0.083	0.001
4	0.7	0.157	0.001
5	0.9	0.265	0.002

### 3.2 Ultrasound: Intra- and inter reader agreement

An ICC of 0.9993 was found for intra-reader agreement and an ICC of 0.9996 was found for inter reader agreement. The data that was used for this calculation can be found in ***S5 Appendix.***

### 3.3 CT reconstruction and postprocessing time

The reconstruction of one dataset, containing 60 CTA scans acquired during three pulsation cycles, took approximately 21 minutes, 51 minutes and 180 minutes for Hybrid-IR, DLR and MBIR, respectively. The image registration algorithm took approximately 30 minutes for one dataset.

### 3.4 Diameter change pattern

**Fig 3:**
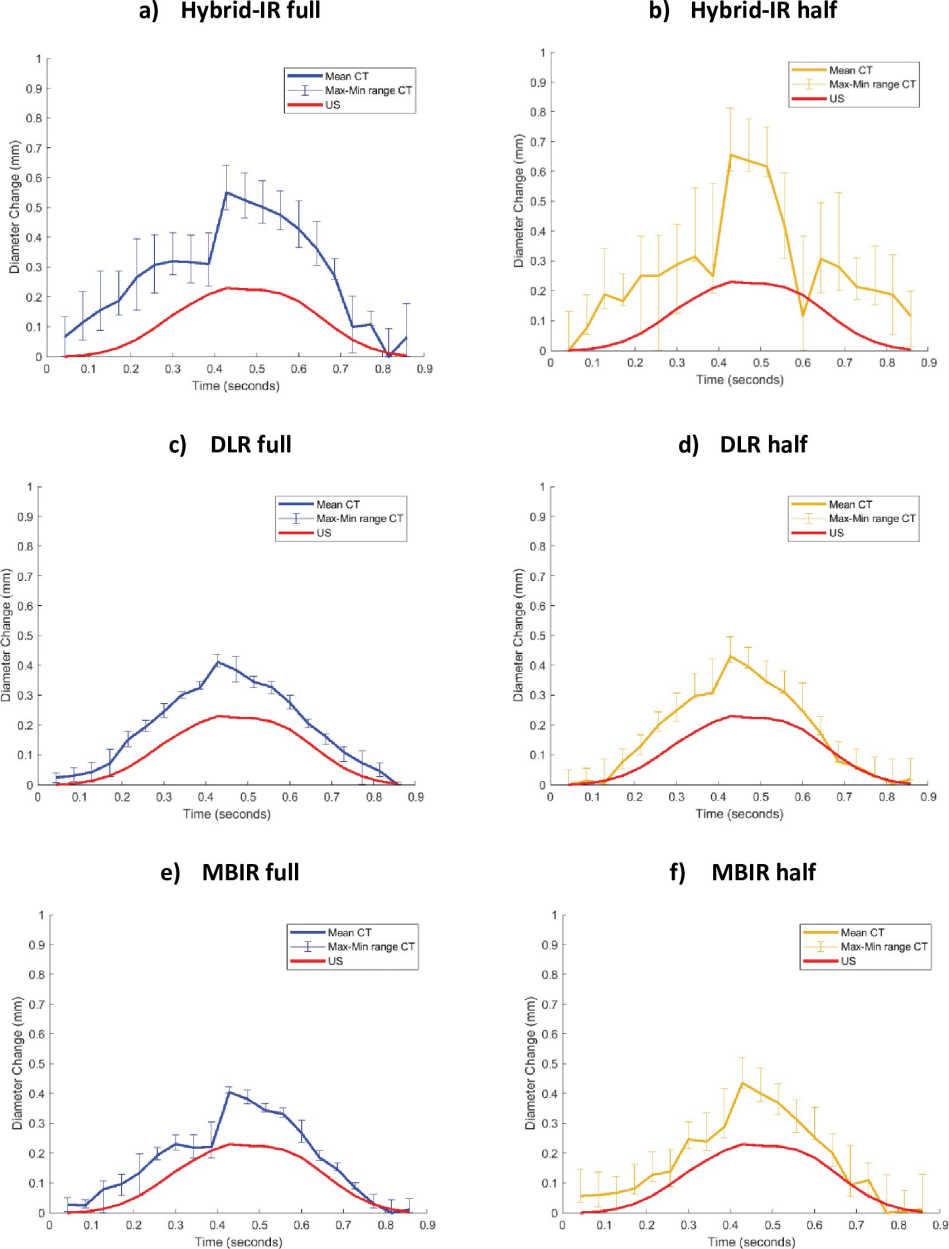
Averaged diameter change pattern over three pulsation cycles with the min-max range of every time phase for 4D CTA and the diameter change pattern of US as a reference. Flow amplitude = 0.9 L/min. Reconstruction methods: Hybrid Iterative Reconstruction (Hybrid-IR)–Full (a) and Half (b), Deep Learning Based Reconstruction–Full (c) and Half (d), Model-based iterative reconstruction (MBIR)–Full (e) and Half (f).

The diameter changes over three pulsation cycles captured with the three image reconstruction algorithms is shown in **Fig 3.** Hybrid-IR exhibited greater variability between the pulsation cycles and a larger disparity with US, compared to DLR and MBIR. The variability between pulsation cycles was reduced when employing the full reconstruction mode as opposed to the half reconstruction mode. 4D CTA captured a diameter change pattern comparable to the pattern captured by US. The data that was used for this visualization can be found in ***S6 Appendix.***

### 3.5 Difference between 4D CTA and US measurements

**Fig 4:**
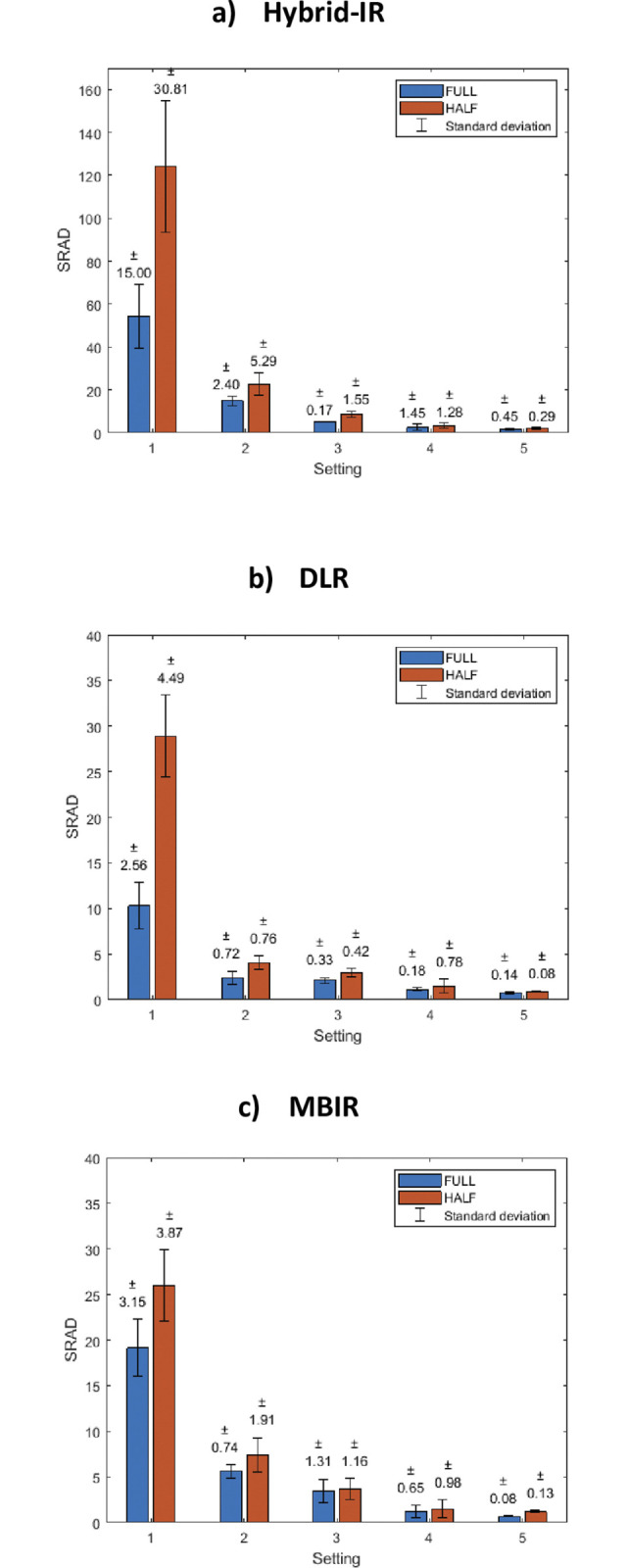
**SRAD for Hybrid IR (a), DLR (b) and MBIR (c), both half and full reconstruction mode.** The error bars represent the standard deviation. Please be aware that the scale of the Y-axis in panel a differs from that in panels b and c.

For the three reconstruction methods, the SRAD for half and full reconstruction mode is shown in **Fig 4**. The SRAD and standard deviation decreased as the flow amplitude setting increased, regardless of the reconstruction method that was used. Hybrid-IR exhibited a higher SRAD and a greater standard deviation in comparison to DLR and MBIR. Full reconstruction mode exhibited diminished SRAD and lower standard deviations, compared to half reconstruction mode. A table with the SRAD values and their standard deviations are shown in ***S7 Appendix***.

## Discussion

### 4.1 Key findings

The SRAD outcomes were comparable for the DLR and MBIR algorithms and both algorithms resulted in a lower SRAD as compared to the Hybrid-IR algorithm for every pump setting. Previous studies found good image quality, high-contrast spatial resolution and good noise reduction for MBIR and DLR algorithms compared to the Hybrid-IR algorithm, which is in agreement with our study. [19–22] An important addition is that the processing time of DLR is significantly shorter than MBIR (51 versus 180 minutes). Full reconstruction mode exhibited in diminished differences with US and lower standard deviations, compared to half reconstruction mode, which is in line with a previous study [23].

Increasing pump amplitudes led to an increase in dilation of the silicon tube, which led to a reduction of SRAD, regardless of the image reconstruction method that was used. This aligned with expectations, since larger diameter changes are more easily captured with 4D CTA. Even sinusoidal expansions of diameter changes smaller than the CT detector element size of 0.25 mm were observed. Differences between 4D CTA and US may be the result of noise and other disturbances like partial scan artifacts [24]. The impact of these disturbances intensifies when attempting to capture smaller diameter changes. Ensuring a good signal-to-noise is crucial, given the ultimate objective of detecting sub-millimeter vessel motions, especially when applied to the cerebral vessels.

### 4.2 Strengths

Our study provides a solid foundation for optimal image reconstruction, which enables further exploration of the possibilities of 4D CTA to capture vessel motion. Another strength is that reliable reference US measurements of the diameter changes were used. The standard deviation of US is shown to be in the order of magnitude of 10^−3^ and the intra–and inter reader agreement is excellent with an ICC over 0.99. The sampling frequency of US is sufficient to prevent aliasing for 70 bpm motions. In addition, a deformable registration based on normalized gradient field was selected for postprocessing. This technique relies on image gradients rather than intensities, thereby diminishing the influence of intensity differences within the tube and omitting the need for stringent thresholds to identify the tube’s edges. Final, three pulsation cycles were scanned with 4D CTA, whereas clinical studies in this field often assess only a single cycle. [25–27] Scanning multiple pulsation cycles is crucial to determine the repeatability and accuracy of the motion measurements.

### 4.3 Limitations

The study has several limitations. First, the temporal resolution was not evaluated in this study. Temporal limitations could pose a challenge, when attempting to capture high frequency movements. The minimum gantry rotation time of the CT is 0.28 seconds, which results in temporal overlap of the CTA reconstructions. The extent of overlap depends on the heart rate and the reconstruction mode used. For half reconstruction mode, there is reduced data overlap in comparison to full reconstruction mode, leading to a higher temporal resolution. However, half reconstruction mode results in more artifacts and noise due to the reduced amount of information used for reconstruction [23]. It can be insightful to investigate the temporal limitation of the 4D CTA for full and half reconstruction.

Second, this study evaluates one set of CT acquisition settings and one postprocessing method. Variation in tube parameters could provide better signal to noise ratios, but could result in an increased radiation dose, which is of relevance for clinical implementation. Next to the acquisition parameters, the different FOV sizes for the reconstruction algorithms may have affected the comparison.[23] For the image registration postprocessing method, the edge parameter and regularization parameter were chosen manually. It was anticipated that their influence would be minimal, however the precise effect of these parameters was not quantified in this study.

Moreover, several assumptions had to be done in order to perform a practical phantom experiment. For instance, the attenuation was approximated to the in vivo situation as closely as feasible, through the utilization of water and a human skull, although disparities relative to the in vivo situation may exist. In addition, for this experiment the expansion over 1 cm segment is averaged, assuming a homogeneous expansion over the phantom. Thereby, the orientation of the tube was limited to a single direction and close to the gantry center, whereas in vivo, motions occur in multiple directions and locations of the scanner. The in vivo situation also differs in vessel diameters, vessel wall thickness, configuration of vessels, mechanical properties and flow patterns.

Final, US and 4D CTA were not measured simultaneously, which may have led to a small difference in the measured locations between US and 4D CTA. Moreover, the variability across the pulsation cycles or in between experiments could have been underestimated since the phantom was not moved between measurements resulting in the same position of the phantom relative to the detection elements.

### 4.4 Future perspectives

The next step in the exploration into the possibilities of the 4D CTA to capture vessel wall motion should be determining the spatial and temporal limitations, while using the optimal reconstruction method as provided in this study. Given that the ultimate objective is to capture sub-millimeter vessel wall motion, it is essential to ensure a good signal-to-noise ratio. Both acquisition parameters (tube voltage or tube current), as well as patient characteristics (e.g. heart rate) should be taken into account.

In vivo validation of vessel wall motion is crucial to ensure that actual vessel wall diameter changes are measured rather than imaging noise, or other causes of random or structural errors in the measurements. Superficial arteries outside the cranial region, for example the radial artery in the wrist, could be used for in vivo validation where ultrasound can be used as a reference metric. For intracranial vessels, it is not possible to use ultrasound as a reference metric, due to the attenuation of the skull. This study may therefore serve as a basis for future studies concerning the validation and optimization of 4D CTA for measuring vessel wall pulsations.

## 5. Conclusion

4D CTA can capture a diameter change pattern comparable to the pattern captured by US. The DLR and MBIR algorithms show more accurate results than the Hybrid-IR algorithm. Reconstruction with DLR is >3 times faster than using MBIR. Full reconstruction mode is more accurate than half reconstruction mode.

## Supporting information

S1 Appendix Ultrasound reflection software. (PDF)

S2 Appendix Mask threshold. (PDF)

S3 Appendix Ultrasound diameter. (PDF)

S4 Appendix Data ultrasound maximal diameter changes. (PDF)

S5 Appendix Data for reader variability. (PDF)

S6 Appendix Data for diameter change pattern plots. (PDF)

S7 Appendix Sum of the Relative Absolute Differences (SRAD). (PDF)
