# Supplementary material for: Dynamic Computed Tomography Angiography for capturing vessel wall motion: A phantom study for optimal image reconstruction
Source: PLoS One. 2023 Dec 22;18(12):e0293353. doi: 10.1371/journal.pone.0293353 (PMC10745207; doi:10.1371/journal.pone.0293353)
Supplement: S1 Appendix — (PDF) [file pone.0293353.s001.pdf]

S1 Appendix. Ultrasound reflection software

The tool first demands the user to select a certain image line in an echogram (see top-left panel, **Figure A1**). Next, the software visualizes the image data for this line as a function of time in a so-called M-mode view (see top-right panel). In this M-mode, the user selects the reflections corresponding to the wall-water interfaces in a single timeframe. Next, the motion of these reflections over time is automatically determined by a 2D normalized cross-correlation-based algorithm, so-called echo- tracking, and diameter curves and diameter changes over time are derived. The mean, minimal and maximal diameter change per pump cycle was calculated to determine the expansion of the tube corresponding to the applied pump amplitudes. The 2D kernel and template sizes for echo-tracking were 600 x 600 μm^2 and 1200 µm axially x 1000 µm laterally, respectively. To track the displacementsat subsample level, bilinear interpolation of the RF data was performed and the normalized cross- correlation peak was interpolated by fitting a 2D parabola [13].

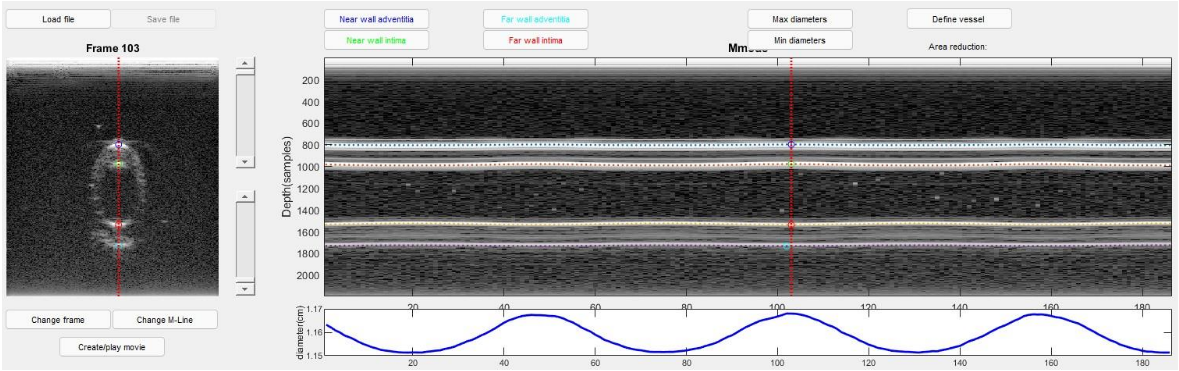

Figure A1. *Analysis of raw radiofrequency ultrasound data, used to estimate the diameter change of the elastic tube lumen. Left panel: manual selection of reflections at the interfaces between lumen and wall. Right panel (top): an image showing the ultrasound data of the red image line over time (so-called M-mode). The tracked reflections are indicated by colored traces. Right panel (bottom): diameter change over time derived from the tracked reflections over time for the outer wall-lumen interfaces.*
