# Supplementary material for: Dynamic Computed Tomography Angiography for capturing vessel wall motion: A phantom study for optimal image reconstruction
Source: PLoS One. 2023 Dec 22;18(12):e0293353. doi: 10.1371/journal.pone.0293353 (PMC10745207; doi:10.1371/journal.pone.0293353)
Supplement: S2 Appendix — (PDF) [file pone.0293353.s002.pdf]

S2 Appendix. Mask threshold

This test was meant to examine the influence of the value of the mask threshold, that was used for determining the mean voxel deformation. Nine thresholds were chosen between 70 and 310 (in the range of the edge of the tube). Table A1 shows the influence of choosing another threshold. The maximal diameter and correlation with ultrasound did show minimal variation, which indicated the minimal influence of the threshold for the mask.

Table A1 Influence of the mask threshold

**Mask threshold**	**Correlation with US**	**Maximal diameter CT**
70	0.99	0.04
100	0.99	0.04
130	0.99	0.04
150	0.99	0.04
180	0.99	0.04
210	0.99	0.05
240	0.99	0.05
270	0.99	0.05
310	0.99	0.05
