# Supplementary material for: Dynamic Computed Tomography Angiography for capturing vessel wall motion: A phantom study for optimal image reconstruction
Source: PLoS One. 2023 Dec 22;18(12):e0293353. doi: 10.1371/journal.pone.0293353 (PMC10745207; doi:10.1371/journal.pone.0293353)
Supplement: S3 Appendix — (PDF) [file pone.0293353.s003.pdf]

S3 Appendix. Ultrasound diameter

A.

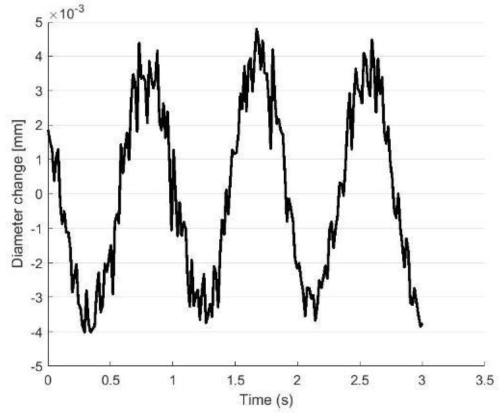

B.

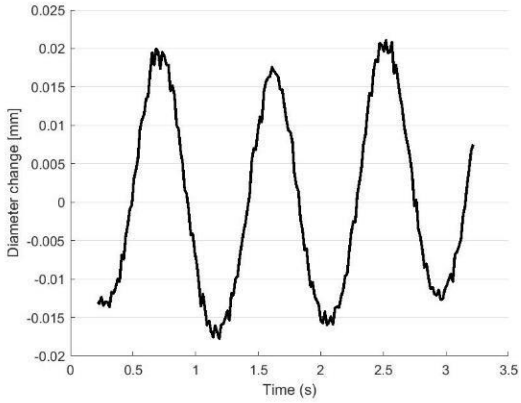

C.

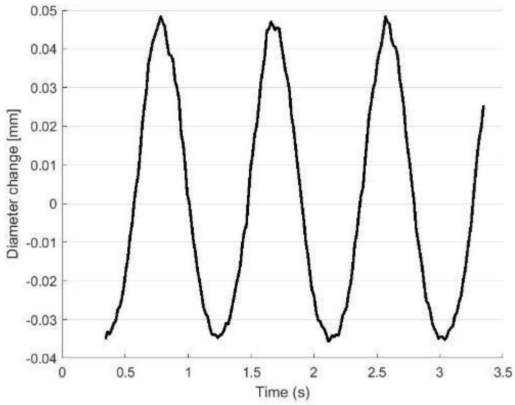

D.

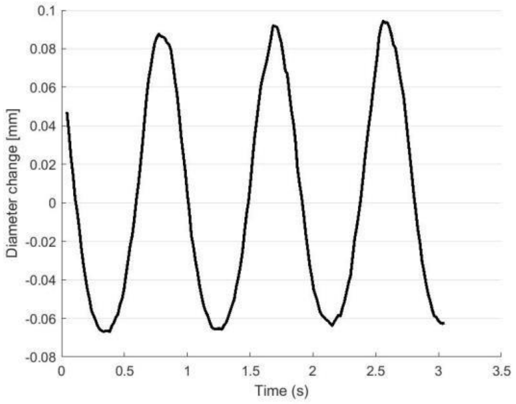

E.

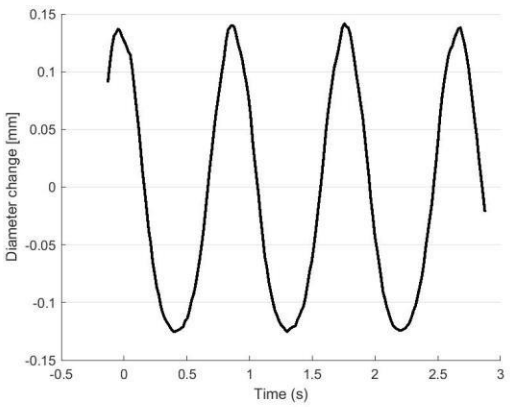

Figure C1. *Ultrasound (US) diameter change for increasing flow wave amplitudes. A. 0.1 L/min, B. 0.3 L/min, C. 0.5 L/min, D. 0.7 L/min, E. 0.9 L/min*
