# Supplementary material for: Dynamic Computed Tomography Angiography for capturing vessel wall motion: A phantom study for optimal image reconstruction
Source: PLoS One. 2023 Dec 22;18(12):e0293353. doi: 10.1371/journal.pone.0293353 (PMC10745207; doi:10.1371/journal.pone.0293353)
Supplement: S4 Appendix — (PDF) [file pone.0293353.s004.pdf]

# S4 Appendix. Data ultrasound maximal diameter changes

| Setting | Pump amplitude | Max. diameter change peak 1 (mm) | Max. diameter change peak 2 (mm) | Max. diameter change peak 3 (mm) | Mean diameter change (mm) | Standard deviation (mm) |
|---|---|---|---|---|---|---|
| 1 | 0.1 | 0.008 | 0.009 | 0.008 | 0.008 | 0.000 |
| 2 | 0.3 | 0.030 | 0.039 | 0.036 | 0.035 | 0.004 |
| 3 | 0.5 | 0.082 | 0.084 | 0.083 | 0.083 | 0.001 |
| 4 | 0.7 | 0.155 | 0.158 | 0.158 | 0.157 | 0.001 |
| 5 | 0.9 | 0.262 | 0.267 | 0.266 | 0.265 | 0.002 |
