# Supplementary material for: Dynamic Computed Tomography Angiography for capturing vessel wall motion: A phantom study for optimal image reconstruction
Source: PLoS One. 2023 Dec 22;18(12):e0293353. doi: 10.1371/journal.pone.0293353 (PMC10745207; doi:10.1371/journal.pone.0293353)
Supplement: S5 Appendix — (PDF) [file pone.0293353.s005.pdf]

S5 Appendix. Data for reader variability

Setting	Diameter Reader 1- 1	Diameter Reader 1- 2	Diameter Reader 2-1	Diameter change Reader 1-1	Diameter change Reader 1-2	Diameter change Reader 2-1
1	11.543	11.578	11.325	0.009	0.012	0.011
2	11.623	11.531	11.387	0.039	0.034	0.035
3	11.525	11.562	11.417	0.084	0.089	0.089
4	11.537	11.583	11.415	0.161	0.167	0.163
5	11.600	11.517	11.432	0.267	0.264	0.267
