# Supplementary material for: Dynamic Computed Tomography Angiography for capturing vessel wall motion: A phantom study for optimal image reconstruction
Source: PLoS One. 2023 Dec 22;18(12):e0293353. doi: 10.1371/journal.pone.0293353 (PMC10745207; doi:10.1371/journal.pone.0293353)
Supplement: S6 Appendix — (PDF) [file pone.0293353.s006.pdf]

S6 Appendix. Data for diameter change pattern plots

DLR	Beat 1	Beat 2	Beat 3
FULL	0.0394973143065402	0.0177229415223379	0.00728176703655770
	0.0565449887202307	0.0130627812786743	0.0114615787396599
	0.0765195930858997	0.00913357288290007	0.0504171730662062
	0.118721578771033	0.0265515435111419	0.0694185033096306
	0.180461917860175	0.127730936344530	0.137484667402268
	0.215082689554686	0.181995750250608	0.178971931789242
	0.272810974787128	0.226177483218430	0.226107776383988
	0.313869328059615	0.295134819989626	0.290861518239867
	0.321123513550723	0.323369919727767	0.346079506408452
	0.421932099331959	0.394632260148486	0.437773594269271
	0.412649187601868	0.345393281181565	0.430179872295648
	0.362627409380674	0.326844121284682	0.356660370912518
	0.347317167692216	0.305945842891442	0.338400478187932
	0.300512441756318	0.253466092755150	0.272611987875477
	0.219111391749649	0.192254521912605	0.220261181994561
	0.171437088813100	0.161633334114064	0.135814092000239
	0.127848448898726	0.103053388383358	0.0784024084082802
	0.112934842034822	0.0607800441966622	0
	0.0746160785411210	0.0264518386392343	0.0263663785740831
	0	0	0.00791084267419029
**DLR**	**Beat 1**	**Beat 2**	**Beat 3**
**HALF**	0.0488585028123656	0.0182157330180743	0.0257369554866260
	0.0426071067077327	0.0456850660028518	0.0555463372832765
	0.0843801203003247	0	0.0151484580562111
	0.119870787024365	0.0993726369540871	0.103181043556753
	0.164364518160229	0.157020136956643	0.166506729865541
	0.244008006756296	0.241512808305624	0.179391766795438
	0.307087117407587	0.280477447429042	0.214421831985911
	0.299587335573579	0.373074226717056	0.290974724308917
	0.301159005073719	0.356196163227616	0.421498413831543
	0.452560380894849	0.466572884901437	0.496282649893431
	0.399054749286034	0.461251110631995	0.424246671200690
	0.339399193342074	0.416565996755995	0.387075938091115
	0.311672693957554	0.361698792499172	0.382550756436255
	0.248442340946986	0.294656841295748	0.341294384673803
	0.225705033374013	0.179364251323539	0.228206492590797
	0.145264211626235	0.0760426664167690	0.106539310361737
	0.0728097952818430	0.119675195606916	0.0770030908508512
	0.0473963854557358	0.0932287410528621	0.0264484100777773
	0	0.0867544219520733	0
	0.0201737791626178	0.0882734092395698	0.0291845588478346

Hybrid-IR FULL	Beat 1	Beat 2	Beat 3
	0.133879422802510	0.0639009000575275	0
	0.217091008038772	0.0760642257426225	0.0542299618081965
	0.287059670115144	0.0943836961836975	0.0884257991161519
	0.287492737888762	0.139514886947785	0.158742240808079
	0.395320275823301	0.217218800639951	0.156566807620999
	0.407781812141104	0.283117092680680	0.214550565629923
	0.415806504703664	0.273108174610526	0.307765042957566
	0.406345028047270	0.247379423849108	0.391789354488699
	0.395207023902218	0.236412974466380	0.414763799526132
	0.642045366429522	0.492896636838496	0.587369136756670
	0.615570981243405	0.465373290910645	0.567683893519304
	0.589889423387948	0.446851171475309	0.536710292126581
	0.555292274288672	0.425646707325609	0.515578886010399
	0.523237115889883	0.365864077176639	0.465657084591866
	0.452906355802210	0.305723957874335	0.396243242006447
	0.327646422373846	0.257965435789174	0.263092027150995
	0.0116271621691766	0.202439938152856	0.180779150920596
	0.103603528596954	0.140809524547194	0.151485617643623
	0	0.0113899831902309	0.0952199465070103
	0.178713842163738	0	0.0514227371908804
**Hybrid-IR HALF**	**Beat 1**	**Beat 2**	**Beat 3**
	0	0.0754552962580668	0.130510076020041
	0.181678886064824	0.0546465094879189	0.185358425768525
	0.340469480649726	0.139554479206785	0.245139553817277
	0.257012440681372	0.179997601613024	0.208830869197660
	0.382233709380722	0.305376235530339	0.0551096121439976
	0.320424032214837	0.385975321962111	0
	0.423736322185775	0.330035058906517	0.124496053172349
	0.347580454389163	0.328826726174851	0.544040383174924
	0.326164384594392	0.192700966552508	0.559993494390345
	0.783395113769418	0.603308559243938	0.812068979388874
	0.755616954121703	0.598146166466369	0.776701446001615
	0.736184260260495	0.582187742864508	0.748528446796639
	0.595162621504584	0.306902477967466	0.577816238412205
	0.265192760196542	0	0.384424386608064
	0.495878735362010	0.193864239700947	0.456863702137491
	0.400322934128650	0.201577896746343	0.530341130738936
	0.260259190910592	0.254834538680866	0.309361114251514
	0.154394641200755	0.350150604343025	0.255017864400458
	0.217280004583571	0.320029759900200	0.0532095343094907
	0.184447416151401	0.199898055777501	0.0120325909960672

MBIR FULL	Beat 1	Beat 2	Beat 3
	0.0510315059499833	0.0324644618862169	0.00476885314029785
	0.0431530345596376	0.0278758290245884	0.0155463677306433
	0.107871455027644	0.0954814690109274	0
	0.129606202346062	0.0930333208032153	0.0589554237205125
	0.196985068560778	0.114108903158094	0.0587980208108951
	0.218823825295387	0.190359689404804	0.178795443146873
	0.225381644756133	0.260363623515763	0.210525574981919
	0.183431953441371	0.262803899336526	0.244723939804286
	0.183612708526535	0.247103693246553	0.305541110715355
	0.423098584451449	0.401090332118743	0.413767522958761
	0.412508754137353	0.364859614679276	0.394021682939083
	0.367421781004256	0.338431281959879	0.341621687241491
	0.352979120293615	0.331580975414965	0.320879556646285
	0.311339127594568	0.236274378984520	0.290273611595238
	0.208947154953463	0.174342551323363	0.202381182553866
	0.167440949300681	0.142815712144162	0.141387012508118
	0.0975874526531806	0.0805488614953775	0.100974888200007
	0.0623178703806482	0.0231491580938470	0.0156322281249821
	0.00313058587674275	0	0.0431147416257294
	0	0.0278456385156844	0.0468537339567443
MBIR HALF	Beat 1	Beat 2	Beat 3
	0.128731054253339	0.0779899975516463	0.146957423776424
	0.116339850890588	0.100800847673050	0.137586156676202
	0.123000077786448	0.117513991640277	0.118663955988021
	0.163656549349231	0.0997585244838173	0.150643379727167
	0.205346679023815	0.150592728061698	0.198101511520623
	0.187553666958944	0.189540567710278	0.212387092838020
	0.302675826737204	0.306339669396225	0.276665806697849
	0.302338018664161	0.269444774671015	0.336292869182215
	0.325227011402987	0.334811520111420	0.417717105719928
	0.520688746588878	0.454212024533571	0.510453828497179
	0.484685351271972	0.425680732173261	0.455035961863979
	0.431918086546665	0.420076207221327	0.407809113988638
	0.374294668896971	0.356958409131729	0.378788406526729
	0.353474095929394	0.246259749432831	0.348790822452365
	0.245370289686850	0.265573867479698	0.246406167550755
	0.132265607981215	0.134562041662323	0.224534438954034
	0.166909976608542	0.160457264886189	0.160025889356983
	0.0801191984833394	0	0.128760036962254
	0.0753957530024474	0.0609885665502952	0
	0	0.107390706562733	0.130017352887555

**Ultrasound**
0.000459601324538490
0.00377176583421221
0.0132677095856675
0.0301985687201027
0.0575194027953705
0.0959646719813571
0.139424769697317
0.176011349474916
0.208819931433352
0.229943915771087
0.225512209830141
0.223178322741622
0.211595454048419
0.185670157514057
0.142122564985530
0.0960281050457500
0.0560816174629602
0.0287600569236643
0.0116324159042589
0.00336056223585259
