# Supplementary material for: Dynamic Computed Tomography Angiography for capturing vessel wall motion: A phantom study for optimal image reconstruction
Source: PLoS One. 2023 Dec 22;18(12):e0293353. doi: 10.1371/journal.pone.0293353 (PMC10745207; doi:10.1371/journal.pone.0293353)
Supplement: S7 Appendix — (PDF) [file pone.0293353.s007.pdf]

S7 Appendix. Sum of the Relative Absolute Differences (SRAD)

Flow amplitude	Beat 1	Beat 2	Beat 3	Mean	STD
**Hybrid-IR - full**					
**0.1 L/min**	36.94	73.54	52.41	54.30	15.00
**0.3 L/min**	17.12	16.00	11.57	14.90	2.40
**0.5 L/min**	5.31	5.44	5.02	5.26	0.17
**0.7 L/min**	4.62	1.78	1.36	2.59	1.45
**0.9 L/min**	2.30	1.22	1.62	1.71	0.45
**Hybrid-IR - half**					
**0.1 L/min**	167.46	107.91	97.54	124.30	30.81
**0.3 L/min**	19.30	30.12	18.54	22.65	5.29
**0.5 L/min**	6.76	8.97	10.53	8.75	1.55
**0.7 L/min**	4.42	1.53	4.03	3.33	1.28
**0.9 L/min**	2.47	1.81	2.36	2.21	0.29
**DLR - full**					
**0.1 L/min**	13.81	9.32	7.76	10.30	2.56
**0.3 L/min**	1.92	3.44	1.90	2.42	0.72
**0.5 L/min**	2.09	1.71	2.52	2.11	0.33
**0.7 L/min**	1.34	1.12	0.91	1.12	0.18
**0.9 L/min**	0.94	0.60	0.72	0.75	0.14
**DLR - half**					
**0.1 L/min**	33.25	22.73	30.78	28.92	4.49
**0.3 L/min**	4.84	3.03	4.27	4.05	0.76
**0.5 L/min**	3.42	2.42	3.11	2.98	0.42
**0.7 L/min**	2.55	1.20	0.71	1.48	0.78
**0.9 L/min**	0.81	1.00	0.87	0.90	0.08
**MBIR - full**					
**0.1 L/min**	19.52	15.09	22.79	19.13	3.15
**0.3 L/min**	6.08	6.23	4.59	5.64	0.74
**0.5 L/min**	1.61	4.20	4.54	3.45	1.31
**0.7 L/min**	2.10	0.98	0.56	1.21	0.65
**0.9 L/min**	0.79	0.63	0.61	0.67	0.08
**MBIR - half**					
**0.1 L/min**	25.83	21.34	30.83	26.00	3.87
**0.3 L/min**	5.65	10.05	6.49	7.40	1.91
**0.5 L/min**	2.05	4.55	4.49	3.70	1.16
**0.7 L/min**	2.88	0.86	0.74	1.50	0.98
**0.9 L/min**	1.21	1.03	1.34	1.19	0.13
